# Knowledge of rabies and dog-related behaviors among people in Siem Reap Province, Cambodia

**DOI:** 10.1186/s41182-018-0102-0

**Published:** 2018-06-08

**Authors:** Sothy Sor, Michiyo Higuchi, Mohammad Abul Bashar Sarker, Nobuyuki Hamajima

**Affiliations:** 1 Siem Reap Provincial Health Department, Siem Reap, Cambodia; 2 Department of Global and Community Health, Nagoya City University School of Nursing, 1, Kawasumi, Mizuho-cho, Mizuho, Nagoya, 467-8601 Japan; 3 Health Economics Unit, Ministry of Health and Family Welfare, Dhaka, Bangladesh; 4 Department of Healthcare Administration, Nagoya University Graduate School of Medicine, Nagoya, Japan

**Keywords:** Rabies, Post-exposure prophylaxis, Knowledge, Behaviors, Rural population, Cambodia

## Abstract

**Background:**

The rabies incidence and number of dogs in Cambodia are much higher than in nearby countries. Knowledge and behaviors which are related to rabies and/or dogs are considered to be contributing factors for rabies infection control in the community; however, such information in rural Cambodia is limited. This cross-sectional study aimed to assess knowledge and experiences related to rabies as well as dog-related behaviors among people in Siem Reap Province, and to identify the specific factors associated with adequate knowledge.

**Methods:**

Four-stage sampling was employed to identify villages and households. In total, 360 respondents were interviewed using a structured questionnaire. Data were descriptively summarized and logistic regression was performed to estimate odds ratios of adequate knowledge related to rabies for respondents’ characteristics.

**Results:**

Only 9.7% of respondents had adequate knowledge of rabies. Of the respondents, 86.9 and 18.3% had experienced hearing of or seeing a suspected rabid dog and a suspected rabid human, respectively. More than two-thirds (70.6%) of households had at least one dog, and the ratio of dog to human populations was 1: 2.8. Only a few owners had vaccinated dogs, used a cage, or tied up their dog. Visiting a health center was the first choice of treatment for respondents when bitten by a dog. However, post-exposure prophylaxis (PEP) was not commonly expected as a treatment choice by respondents. Those with higher education were more likely to have adequate knowledge than those with no education (adjusted OR 12.34, 95% CI 2.64–57.99, *p* < 0.01). Farmers and non-poor families were also less likely to have adequate knowledge than those of other professions and poor families (adjusted OR 0.30, 95% CI 0.12–0.76, *p* = 0.01, and adjusted OR 0.13, 95% CI 0.04–0.47, *p* < 0.01, respectively).

**Conclusions:**

High dog population, inadequate knowledge of rabies, low recognition of human rabies, and poor dog management were found to be serious challenges for controlling rabies. Health education related to rabies should be introduced, targeting farmers in particular who easily encounter stray dogs but have little knowledge of rabies risk factors and signs. At the same time, PEP delivery and dog management should be improved.

## Background

Rabies is an ancient viral zoonotic disease that can be transmitted to humans from infected animals such as dogs, cats, and other types of wildlife. Among them, dogs are the most important rabies reservoir; 96% of reported human rabies cases are caused by dog bites [1, 2]. It is estimated that over 59,000 people die of rabies annually worldwide and majority of the cases occur in Asia and in Africa [3–8]. Although the fatality rate of rabies infection is nearly 100%, rabies can be prevented by appropriate vaccination of high-risk people in advance (pre-exposure prophylaxis or Pre-EP) and the victims of a dog bite (post-exposure prophylaxis or PEP) [9]. Globally, more than 15 million people worldwide receive PEP annually to prevent rabies deaths [3]. The global community has a strong commitment to eradicating rabies worldwide by the year 2030 [10].

In order to prevent human deaths from rabies, several strategies are implemented, including increasing accessibility to Pre-EP for people at risk and PEP to dog bite victims, and promoting awareness and knowledge related to rabies in the community through on-site health education or mass media. Along with dog registration, dog vaccination against rabies is also needed [3, 11, 12]. To implement the above-mentioned measures, collaboration is required among the public health sector, veterinary health sector, communities, and others. It is recommended that the public health sector strengthen the national rabies policy, including rabies control, and comprehensively coordinate rabies surveillance. It is also important for the veterinary health sector to develop a dog management policy. Communities need to change their attitudes toward and behaviors related to dogs, and development partners should support or facilitate technical and financial processes to all sectors listed above. Public education of rabies is not easy and simple. The willingness to participate in health education and awareness of rabies among people is limited [13]. Health promotion and education is most likely to be successful through the cooperation of human and animal health authorities [14].

Cambodia is one of the countries greatly burdened by rabies [15]. According to a study conducted by the Institute Pasteur of Cambodia, the estimated incidence was 5.8/100,000 (95% CI 2.8–11.5) of the population and 810 human rabies deaths would occur in 2007 (95% CI 394–1,607) in the whole country in 2007. The same study found that the ratio of dog population to human population is 1:3, and by that estimate the dog population in Cambodia could be 5 million. The rabies incidence and number of dogs in Cambodia are much higher than in nearby countries in Southeast Asia. Although the number of rabies patients is smaller than that of other common diseases such as malaria, dengue fever, and acute respiratory infection, the estimated number of deaths were greater than those from other infectious diseases due to the highest fatality rate [16].

Basic knowledge of the disease and its treatment are important for rabies infection control in the community. In addition, personal experience related to rabies and dogs as well as dog-related behaviors such as feeding and managing them are also considered to be contributing factors. Data on the current situations are necessary both for health authorities and communities to improve community knowledge and behaviors. However, such information available in Cambodia is only from Phnom Penh and Kandal Province [17], but not from rural areas. The objectives of this study were to assess knowledge of rabies, experiences related to rabies and dog, and dog-related behaviors among people in Siem Reap Province, and to identify factors associated with the adequate knowledge.

## Methods

### Study design and setting of the study

A cross-sectional study was carried out in Siem Reap Province, Cambodia, from December 24, 2013 to January 13, 2014. Multi-stage cluster sampling was undertaken. First, 3 from among 12 districts in Siem Reap Province were randomly selected. For the second stage, two communes were randomly selected from each district. For the third stage, two villages were randomly selected from each commune. Based on this method, we therefore obtained 12 villages in total from 926 villages within Siem Reap Province. According to the provincial health report in 2013, there were a total of 13,685 people living in the 2383 households in the 12 selected villages. The average population per village was around 1140 (ranging from 582 to 2130 people per village), and the average number of households per village was approximately 198 (ranging from 96 to 375 households per village). Among the 12 villages selected, two were located in a mountainous area but the other 10 villages were in a lowland rice field area. The nearest village was approximately 15 km away from Siem Reap town, while the farthest village was approximately 75 km away. The road was travelable to every village at that time because it was during the dry season.

### Participants

Thirty respondents were chosen from each village. Because households are usually located alongside the main road, the target households were systematically sampled along the road by using a specified interval for each village: the number of households in the village divided by 30. The first households for the fourth stage were selected from either side of the village boundary where the main road passes. When no eligible respondents were available at the selected house, the next house was selected. Only one respondent was interviewed in each target household, and the primary target of the interview in each selected household was the head of the household. If the household head was not available at that time, a member of the household aged 18 years or older was accepted as the respondent. In total, 360 respondents were obtained.

### Data collection

A structured questionnaire was developed based on previous studies [13, 17–19], and modified to suit the local context by consulting with experts. The questionnaire included the following items: (1) characteristics of respondent (age, gender, marital status, education, occupation, monthly income, and dog ownership), (2) knowledge related to rabies, (3) experience related to rabies and dogs, and (4) dog-related behaviors.

Four interviewers were trained by the principal investigator for 2 days before data collection. Health centers provided logistic support and the Village Health Supporting Group (VHSG) guided interviewers in each village. A face-to-face interview was conducted when verbal consent was obtained from an eligible respondent.

### Data entry and analysis

Data analyses were performed in the following steps; first, respondents’ characteristics, knowledge, experience, and behaviors were descriptively summarized. Chi-squared tests were performed to investigate associations between dog ownership and each respondent characteristic. For evaluating the level of rabies-related knowledge, correct replies were counted, then the distribution of the number of correct replies was described. If replies to six knowledge questions were all correct, it was classified as good knowledge, and otherwise as inadequate knowledge. Then logistic regression analyses were performed to estimate crude and adjusted odds ratios (ORs) of good knowledge for respondents’ characteristics, which were mutually adjusted, presented with 95% confidential intervals (CIs). *P* values less than 0.05 were considered statistically significant. Epi-info 7 software program developed by the Center for Disease Control and Prevention (CDC), USA, was used for data entry, data management, and analyses.

### Ethical clearance

Approval for the study was obtained from the National Ethics Committee for Health Research of the National Institute of Public Health, the Cambodian Ministry of Health. The Siem Reap Provincial Health Department, which is the local health authority, provided official permission to conduct research in the area. All respondents were orally informed of the study objectives and procedures. They were also assured that their responses would be kept anonymous and confidential.

## Results

### Socio-demographic characteristics of respondents

Among 360 selected households, the mean number of people per family was 5.4. Of the respondents, 73.3% were females while 26.7% were males. The mean age was 37 years old (ranging from 18 to 84 years). Most of the respondents (81.7%) were currently married. Only 12.5% of the respondents had secondary education or above, while nearly one-third of them had never received any formal education whatsoever. Almost four of five respondents were farmers with or without extra work. The median monthly income per household was about 129 USD (ranging from 50 USD to 1200 USD). More than two-thirds of households had at least one dog (Table 1). The average number of dogs per household was 2.0 (maximum 12) including households without a dog, and 2.8 excluding households without a dog. The total dog population was 704 among 360 households. The ratio of the dog population to the human population was 1: 2.8. There was no evidence of the association between investigated socio-demographic characteristics and dog ownership (Table 2).

**Table 1:** Respondents’ characteristics (*n* = 360)

Variables	Number (*n*)	Percentage (%)
Age group		
< 30 years	102	28.3
30–39 years	85	23.6
40–49 years	73	20.3
≧ 50 years	100	27.8
Gender		
Female	264	73.3
Male	96	26.7
Marital status		
Currently married	294	81.7
Currently unmarried	66	18.3
Education		
No education	107	29.8
Primary	208	57.8
Secondary and above	45	12.5
Occupation		
Farmer	291	80.8
Others^a^	69	19.2
Monthly family income		
< 150 USD	203	56.4
≧ 150 USD	95	26.4
No reply (missing)	62	17.2
Dog ownership		
No	106	29.4
Yes	254	70.6

^a^Others: office staffs, construction workers, teachers, police, soldiers, venders, and others

**Table 2:** Respondents’ characteristics by dog ownership (*n* = 360)

Variables	Total	Not dog owner		Dog owner		*p* value**
	(*N*)	(*n*)	(%)	(*n*)	(%)	
Age group						
< 30 years	102	34	32.1	68	26.8	0.56
30–39 years	85	27	25.5	58	22.8	
40–49 years	73	20	18.9	53	20.9	
≧ 50 years	100	25	23.6	75	29.5	
Gender						
Female	264	76	71.7	188	74.0	0.65
Male	96	30	28.3	66	26.0	
Marital status						
Currently married	294	81	76.4	213	83.9	0.10
Currently unmarried	66	25	23.6	41	16.1	
Education						
No education	107	34	32.1	73	28.7	0.42
Primary	208	56	52.8	152	59.8	
Secondary and above	45	16	15.1	29	11.4	
Occupation						
Farmer	291	84	79.2	207	81.5	0.62
Others^a^	69	22	20.8	47	18.5	
Monthly family income						
< 150 USD	203	63	59.4	140	55.1	0.58
≧ 150 USD	95	24	22.6	71	28.0	
No reply (missing)	62	19	17.9	43	16.9	
District						
Banteay Srey	120	30	28.3	90	35.4	0.41
Kralanh	120	39	36.8	81	31.9	
Svay Leu	120	37	34.9	83	32.7	

^a^Others: office staffs, construction workers, teachers, police, soldiers, venders, and others

^b^Chi-squared test

### Knowledge related to rabies

As shown in Table 3 of the six main questions pertaining to knowledge related to rabies, more than 80% of respondents said rabies was a transmittable disease. Among them, nearly all (98.6%) responded that rabies could be transmitted through a dog bite. Less than two-thirds of the respondents knew that rabies could be prevented. Awareness of dog rabies vaccine was much less known than awareness of human rabies vaccine. Although more than two-thirds of respondents answered that rabies is fatal, 21.1% believed that rabies could be cured. Thirty-five (9.5%) people correctly replied to six (all) questions. Eighty (22.2%), 81(22.5%), 72 (20.0%), 36 (10.0%), 29 (8.1%) people correctly replied to 5, 4, 3, 2, and 1 question(s), respectively. Twenty-seven (7.5%) did not give any correct replies.

**Table 3:** Respondents’ knowledge related to rabies (*n* = 360)

Variables	Number (*n*)	Percentage (%)
Is rabies a transmittable disease?		
Yes	289	80.3
No	16	4.4
Do not know/no reply	55	15.3
Is rabies a preventable disease?		
Yes	223	61.9
No	33	9.2
Do not know**/**no reply	104	28.9
Can a human be vaccinated against rabies?		
Yes	225	62.5
No	23	6.4
Do not know**/**no reply	112	31.1
Can a dog be vaccinated against rabies?		
Yes	96	26.7
No	93	28.8
Do not know**/**no reply	171	47.5
Is rabies a fatal disease?		
Yes	258	71.7
No	16	4.4
Do not know/no reply	86	23.9
Is rabies a disease which is easily cured?		
Yes	76	21.1
No	160	44.4
Do not know/no reply	124	34.5

**Fig. 1:**
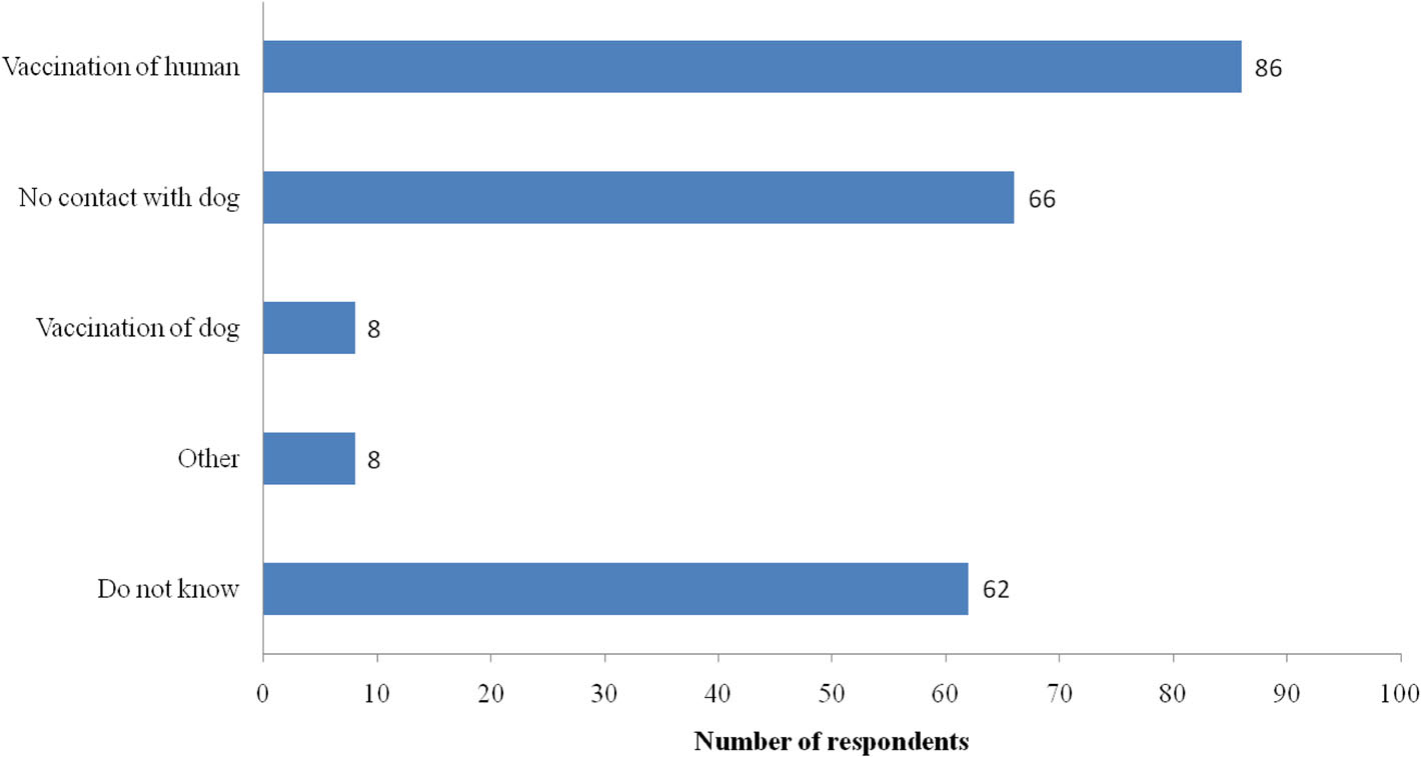
Respondents’ replies about how rabies can be prevented (*n* = 223)

**Fig. 2:**
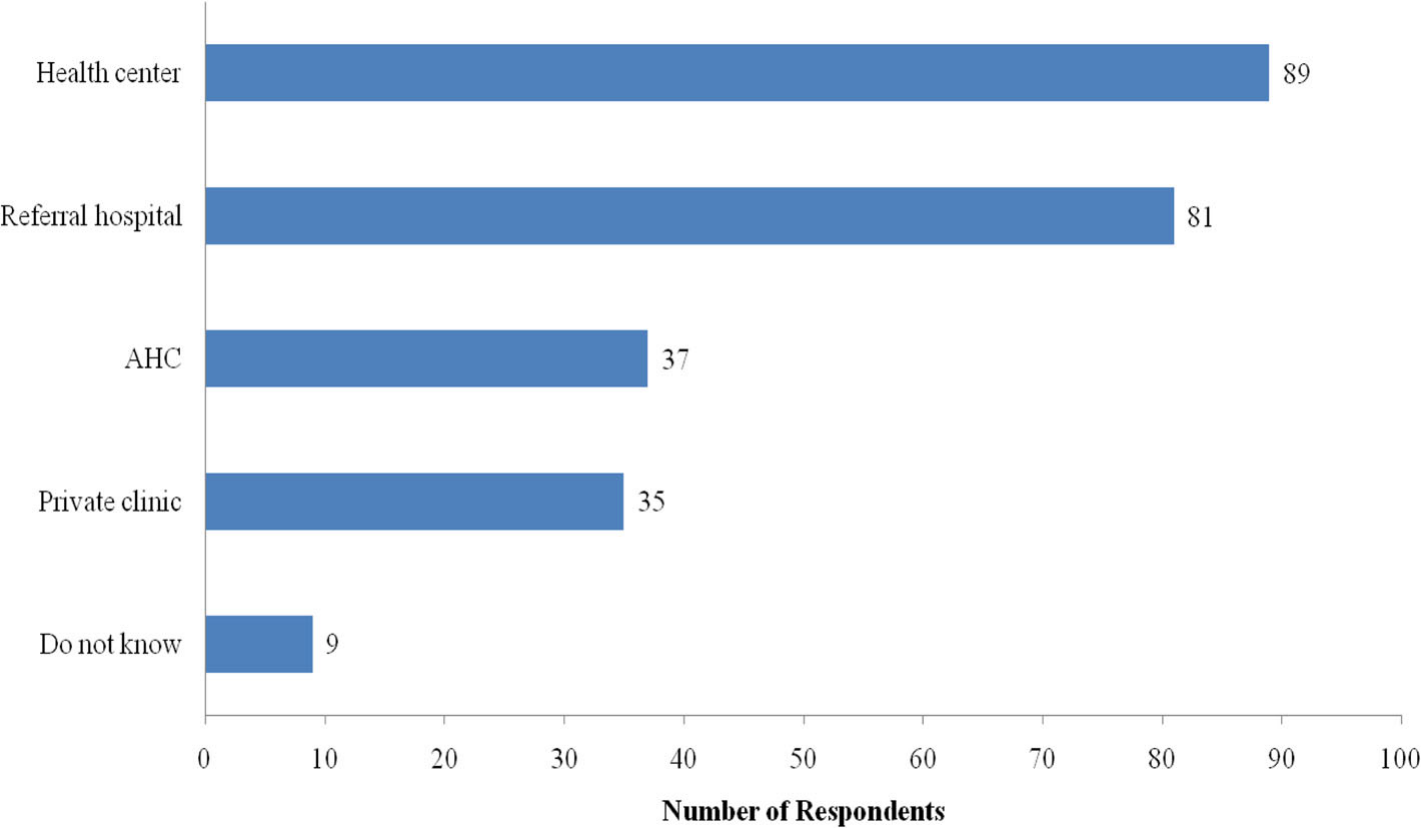
Respondents’ replies about where rabies vaccine is available (*n* = 225). *AHC* Angkor Hospital for Children

Among those who knew that rabies could be prevented, the most frequent responses as to how to prevent were vaccination of humans and no contact with a dog. Only eight respondents suggested vaccinating dogs. Many of them did not know how to prevent rabies (Fig. 1). Of the respondents who knew that a human rabies vaccine was available. The most frequently suggested place where a vaccine was available was health centers (Fig. 2).

### Experience related to rabies and dogs

**Fig. 3:**
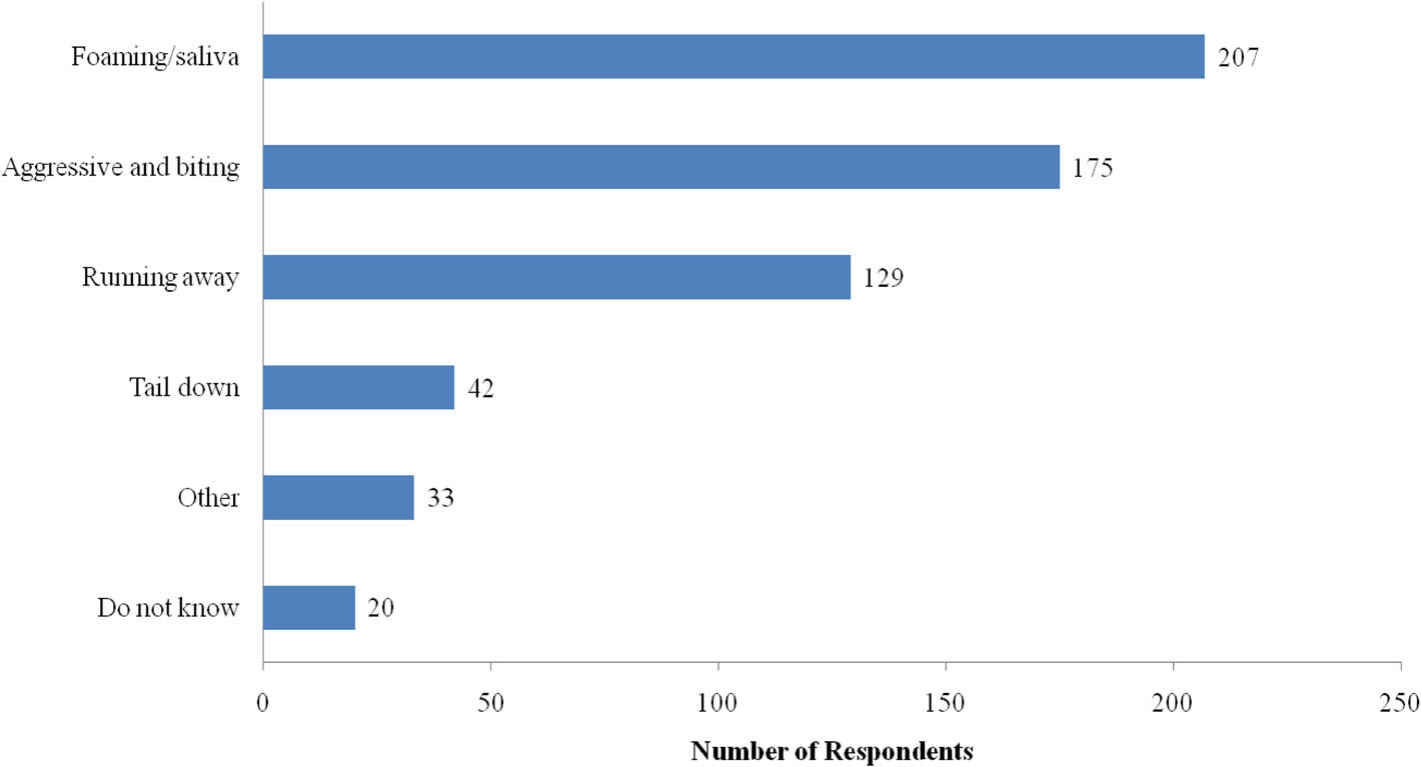
Clinical signs of rabid dogs known by respondents (*n* = 313)

Of the respondents, 86.9% had heard of or seen a suspected rabid dog (Table 4). Among them, 99.4% had heard of suspected rabid dogs from other people. No one answered that TV, radio, newspaper, school, or poster/leaflet were information sources. Figure 3 shows that foaming at the mouth, being aggressive, or biting other dogs or people were frequently suggested by the respondents as clinical signs of suspected rabid dogs. Contrary to the high proportion of those having heard of or having seen a suspected rabid dog, only one-fifth of the respondents had ever heard of or seen human rabies. Nearly half of the respondents (41.9%) had a family member who had been bitten by a dog (Table 4).

**Table 4:** Respondents’ experience related to rabies and dogs (*n* = 360)

Variables	Number (*n*)	Percentage (%)
Have you heard of or seen a rabid dog?		
Yes	313	86.9
No	28	7.8
Do not know/no reply	19	5.3
Have you ever heard of or seen a person with rabies?		
Yes	66	18.3
No	226	62.8
Do not know/no reply	68	18.9
Do you live closely to dogs?		
Yes	338	93.9
No	22	6.1
Do you frequently see stray dogs?		
Yes	234	65.0
No	126	35.0
Have you ever fed stray or roaming dogs?		
Yes	28	7.8
No	332	92.2
Has anyone in your family ever been bitten by a dog?		
Yes	151	41.9
No	205	56.9
Do not know/no reply	4	1.2

### Behaviors related to dog

Among the dog owners, only 44.4% of respondents liked dogs. As for the question, respondents answered that the main purpose of feeding a dog was house security or house protection. Table 5 shows that there were only two households that had already vaccinated their dogs against rabies, and seven households had caged or tied up their dogs. Most of the respondents (84.7%) felt afraid of seeing stray dogs on the road or dogs kept in others’ premises. Nearly a fourth of respondents answered not to seek treatment if bitten by a dog.

**Table 5:** Respondents’ behaviors related to dog

Variables	Number (*n*)	Percentage (%)
Have your dogs been vaccinated against rabies? (*n* = 254)		
Yes	2	0.8
No	252	99.2
Are your dogs kept in a cage or tied? (*n* = 254)		
Yes	7	2.8
No	244	96.0
Do not know/no reply	3	1.2
Do you feel afraid when you see a stray dog? (*n* = 360)		
Yes	305	84.7
No	55	15.3
If you are bitten by a dog, will you seek any treatment? (*n* = 360)		
Yes	276	76.7
No	84	23.3

**Fig. 4:**
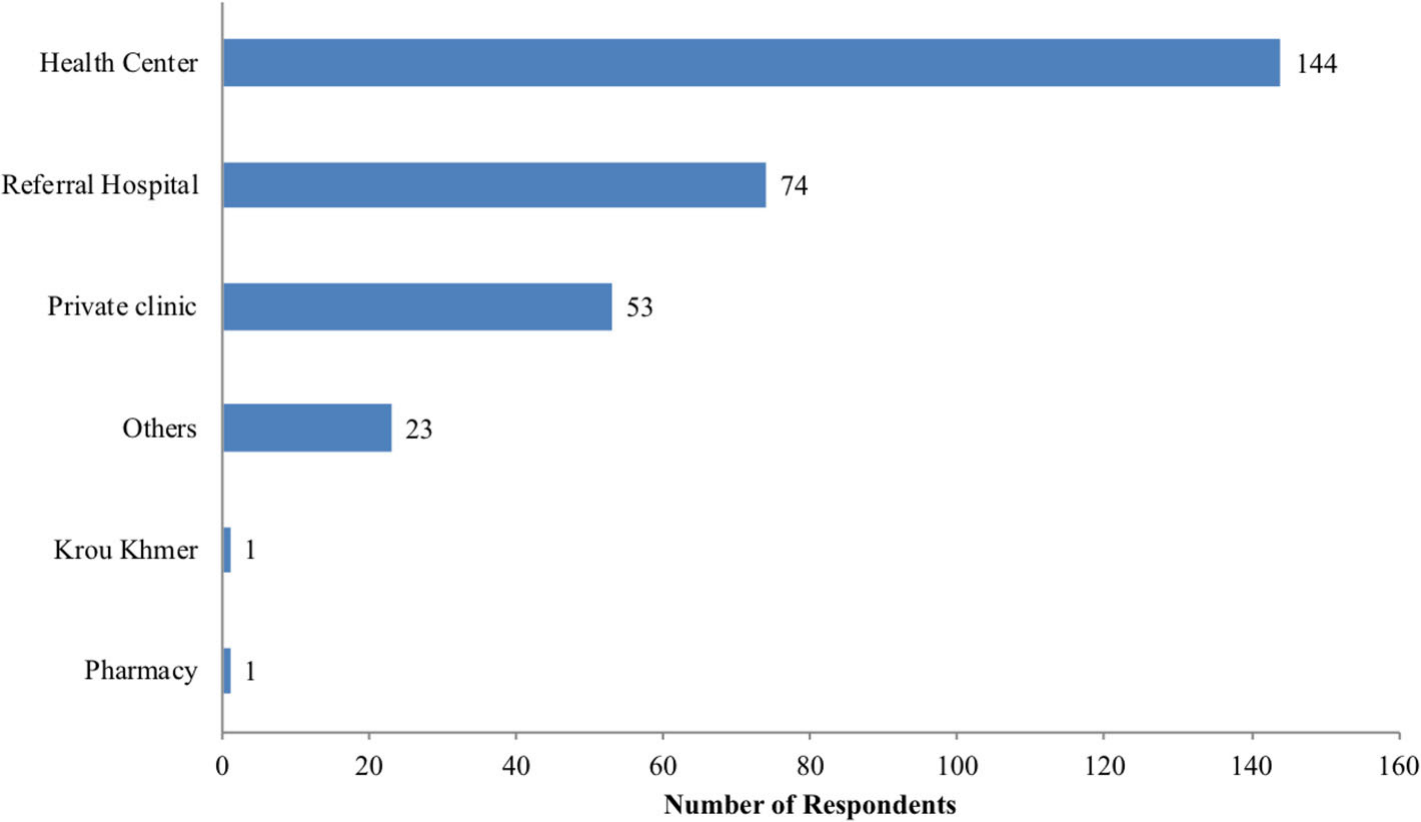
Respondents’ replies about where to find treatment after a dog bite (*n* = 276)

**Fig. 5:**
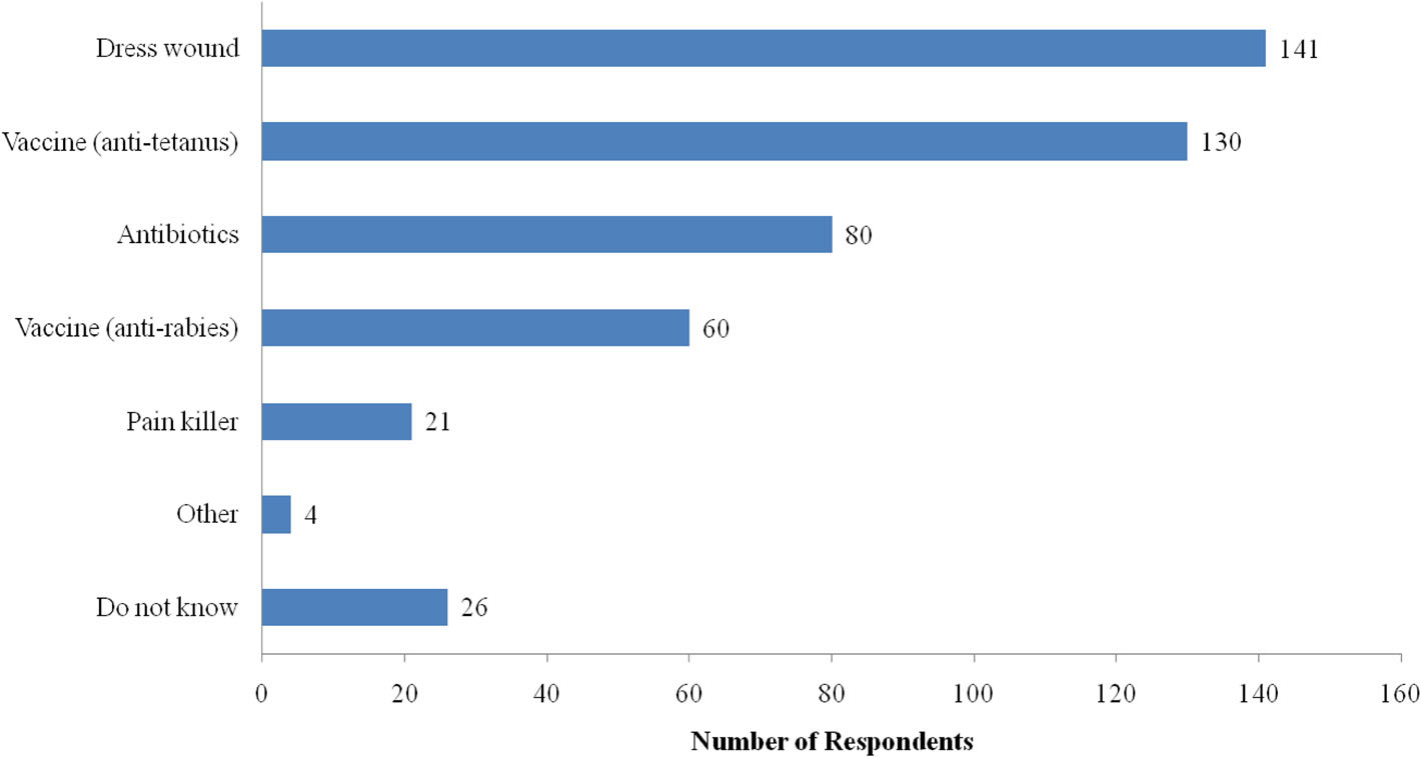
Expected treatment by respondents after a dog bite (*n* = 276)

Among 276 respondents who said they would seek treatment after being bitten by a dog, 114 (52.2%) of them sought treatment at a health center (Fig. 4). Their expected treatment was wound dressing (51.1%), anti-tetanus vaccine (47.1%), antibiotics (29.0%), and anti-rabies vaccine (21.7%), as shown in Fig. 5. Some respondents who did not seek any treatment said they would use a traditional dog bite treatment of sticking rice on the wound. Some respondents would then kill the suspected rabid dog.

### Socio-demographic factors associated with adequate knowledge

Logistic regression indicated that respondents aged 30–39 years were significantly more likely to have adequate knowledge related to rabies than respondents aged less than 30 years (adjusted OR 3.48, 95% CI 1.07–11.42, *p* = 0.04). People with higher education showed statistically greater OR of having adequate knowledge than people with no education (adjusted OR 12.34, 95% CI 2.64–57.99, *p* < 0.01). Farmers and households whose family income was 150 USD or more per month were less likely to have adequate knowledge than the reference group (adjusted OR 0.30, 95% CI 0.12–0.76, *p* = 0.01, and adjusted OR 0.13, 95% CI 0.04–0.47, *p* < 0.01, respectively). (Table 6).

**Table 6:** Crude and adjusted OR of adequate knowledge for respondents’ characteristics (*n* = 360)

Variables	Total	Adequate knowledge		Crude OR		Adjusted OR^b^	
	*n*	*n*	(%)	OR (95% CI)	*p* value	OR (95% CI)	*p* value
Age group							
< 30	102	12	(11.8)	1 (Reference)		1 (Reference)	
30–39	85	13	(15.3)	1.35 (0.58–3.15)	0.48	3.48 (1.07–11.42)	0.04
40–49	73	5	(6.8)	0.55 (0.18–1.64)	0.28	1.46 (0.37–5.85)	0.59
≧ 50	100	5	(5.0)	0.39 (0.13–1.16)	0.09	1.24 (0.29–5.24)	0.77
Gender							
Female	264	25	(9.5)	1 (Reference)		1 (Reference)	
Male	96	10	(10.4)	1.12 (0.51–2.41)	0.79	0.85 (0.32–2.25)	0.75
Marital status							
Currently married	294	27	(9.2)	1 (Reference)		1 (Reference)	
Currently unmarried	66	8	(12.1)	1.36 (0.59–3.15)	0.47	0.09 (0.30–2.76)	0.86
Education							
No education	107	5	(4.7)	1 (Reference)		1 (Reference)	
Primary	208	17	(8.2)	1.82 (0.65–5.06)	0.25	1.85 (0.06–5.76)	0.28
Secondary or above	45	13	(28.9)	8.29 (2.74–25.02)	< 0.01	12.37 (2.64–57.99)	< 0.01
Occupation							
Others^a^	69	16	(23.2)	1 (Reference)		1 (Reference)	
Farmer	291	19	(6.5)	0.23 (0.11–0.48)	< 0.01	0.30 (0.12–0.76)	0.01
Monthly family income							
< 150 USD	203	25	(12.3)	1 (Reference)		1 (Reference)	
≧ 150 USD	95	4	(4.2)	0.31 (0.10–0.93)	0.03	0.13 (0.04–0.47)	< 0.01
Missing	62	6	(9.7)	0.76 (0.29–1.95)	0.57	0.65 (0.21–2.01)	0.45
District							
Banteay Srey	120	13	(10.8)	1 (Reference)		1 (Reference)	
Kralanh	120	18	(15.0)	1.45 (0.68–3.12)	0.34	2.37 (0.95–5.93)	0.06
Svay Leu	120	4	(3.3)	0.28 (0.09–0.89)	0.03	0.44 (0.12–1.54)	0.20
Dog owner							
No	106	8	(7.5)	1 (Reference)		1 (Reference)	
Yes	254	27	(10.6)	1.46 (0.64–3.32)	0.37	1.70 (0.67–4.29)	0.26

^a^Others: office staffs, construction workers, teachers, police, soldiers, venders, and others

^b^Mutually adjusted for all variables listed in the table

## Discussion

This study suggested that more than four-fifths of the respondents knew that rabies was a disease that could be transmitted, and nearly two-thirds said it could be prevented. Although the majority of people knew that it is fatal, some respondents considered it curable. While many respondents had experienced hearing of or seeing a suspected rabid dog but much less people had heard of or seen a suspected rabid human. The dog population was high; however, dog management was still poor. The study found that the respondents with higher education were more likely to have adequate knowledge than those with no education. Farmers and non-poor families were less likely to have adequate knowledge than those of other professions and poor families.

The dog: human ratio in this study was almost the same with the figure in 2007 [16], which implies little intervention had happened for the decade. In Cambodia, dog is called “village security” in the local language, and people keep dogs to protect their houses. According to findings from the same survey, which were not presented in this article, even more than half of the respondents answered “no” to the question which asked if they liked dogs, almost all (97.8%) respondents suggested the purpose of owning a dog was house security (data not presented). It was assumed that house security overweighed a fear of rabies. Or as shown in the results on knowledge related to rabies, people in the community were not aware of severity of rabies compared to the importance of house security.

A study in south-central Bhutan which used similar questions had participants with higher knowledge than our current study [19]. However, the Bhutan study was conducted in a commercial center, and it was assumed that socio-economic status among their respondents was higher than that of our respondents. A post-intervention study conducted in Sri Lanka also demonstrated respondents with higher knowledge than our study after they received health education literature such as leaflets and posters [13]. A study in India conducted in urban slums showed less knowledge than our study in Siem Reap, Cambodia.

The majority of respondents in our study replied that the rabies vaccine was available at health centers or referral hospitals. The national immunization program, however, does not have this vaccine for delivery to health centers and referral hospitals [20]. This misunderstanding might have been caused by those respondents who thought that rabies vaccine was one of the routine immunization vaccines. Most respondents said that they would seek treatment at health centers or referral hospitals after they or their family members were bitten by a dog and they expected that those places would provide them with treatment services. Anti-rabies vaccination was not expected by many respondents. This implied that people were unaware as to the effectiveness and availability of the PEP.

In Cambodian language, the term *Chkai*-*Chkot* implies a dog disease, but there is no specific term for human rabies. To express human rabies, another term to indicate “disease” is usually added before *Chkai*-*Chkot*. This may cause confusion and lead people to think rabies is a disease only among dogs. To avoid the confusion, we asked the respondents separate questions about hearing of or seeing rabies in dogs and humans. Studies in Sri Lanka, south-central Bhutan, and India indicated that a high number of respondents had heard of and seen rabies, for which the question did not specify either dog rabies or human rabies (94.5, 89.6, and 74.1%, respectively) [13, 18, 19]. Findings from this study also had lower proportions than a previous study in Cambodia indicating that 93.2 and 43.5% of respondents had heard of or seen rabies in dogs and human, respectively. Because the previous study in Cambodia was conducted in Phnom Penh and Kandal Province (urban and periurban areas), respondents’ higher education, better living conditions, and accessibility to the PEP center might be possible reasons for the difference in awareness of rabies [17].

In this study, most of the respondents who had heard of or seen a suspected rabid dog knew it from others, but some had seen a suspected rabid dog themselves. This implied that suspected rabid dogs appeared and were well-known in the community. However, rabies cases among humans were not frequently suggested. This might be one of the reasons why respondents were unaware of human rabies and lacked knowledge of rabies among humans. Farmers were less likely to know about rabies than persons in other occupations. They work outside and may frequently encounter stray dogs, some of which might be rabid. Their unawareness might have been due to lack of health information. Therefore, farmers’ knowledge of rabies must be increased. Contrary to our expectations, non-poor families were less likely to have good knowledge scores. In their circumstances, they might pay too little attention to the disease, or they may just have less interaction with animals.

A study of rabies awareness in eight Asian countries (Indonesia, China, India, Philippines, Pakistan, Thailand, Sri Lanka, and Bangladesh) indicated that respondents obtained most of their information pertaining to rabies and its prevention from their relatives or neighbors [21]. The study also suggested that few of the respondents had obtained rabies information from the government authorities of these countries [22]. Although people obtain knowledge related to rabies from relatives or neighbors, sometimes it might be inaccurate or unclear. Public agencies must disseminate precise and practical information related to rabies as much as possible.

There are some limitations in this study. Firstly, we employed systematic sampling along with the main road. It must be valid in Siem Reap situations; however, some houses which were not on the main road might have been missed. Secondly, when no eligible respondents were available at the selected house, and we skipped a house and when the household head was not available, we interviewed somebody else. Although this strategy was practical, it may have caused selection bias. Thirdly, we chose only one person from one household; however, different people might have had different knowledge and experience. Fourthly, we obtained personal experience and behaviors. In addition, we could not have made clear definitions for some terms used in the questionnaire. For example, there was no subjective definition of a “suspected rabid dog” or “stray dog.” These may have caused information bias, including recall bias. Lastly, because face-to-face interviews were employed for the data collections by trained local interviewers in local language, we believe that little misunderstanding due to language would happen. However, some question was difficult to ask people in the community, as discussed before. This may also have caused information bias.

## Conclusions

In conclusion, it was found that there was a high dog population, inadequate knowledge of rabies, low recognition of human rabies, and poor dog management. It was also suggested that although PEP was not available at the health center or referral hospital (public health services), people did not know this. All of these facts could lead to a high rabies burden in Siem Reap province.

Based on the findings of this study, it is recommended that the authorities (provincial, district, commune and village) inform dog owners directly to keep their dog in a cage or tied up, vaccinated, and also to reduce the number of stray dogs in the community. The most effective method of rabies prevention after a dog bite is PEP, which was not known well. The Ministry of Health and National Immunization Program should provide free PEP to dog bite victims through the existing routine vaccination channels. Health education should be developed and disseminated, particularly targeting high-risk groups such as farmers. At the same time, compulsory dog vaccination along with dog registration should also be done to achieve the WHO goal for eliminating rabies by the year 2030.

## Abbreviations

CDC: Center for Disease Control and Prevention
CI: Confident interval
OR: Odds ratio
PEP: Post-exposure prophylaxis
Pre-EP: Pre-exposure prophylaxis
VHSG: Village health supporting group
WHO: World Health Organization
